# Prevalence and risk profiles of metabolic abnormalities (PRIMA): a large-scale, multicenter cross-sectional study among inpatients with schizophrenia in China

**DOI:** 10.1186/s12888-026-07912-6

**Published:** 2026-02-20

**Authors:** Xuemei Liao, Liyun Zheng, Hongyi Li, Xiaobing Lu, Xixiang Mao, Ying Sun, Dongdong Qiao, Yanchi Zhang, Ning Yuan, Chao Li, Yuanjian Yang, Zhiyong Ren, Xiaohong Li, Baoliang Zhong, Gang Wu, Lina Wang, Yunshu Zhang, Jianliang Gao, Yujuan Ma, Yongqing Huang, Guangya Zhang, Yong Qin, Haihang Yu, Cheng Luo, Jiaqi Bao, Hongguang Chen, Tianmei Si

**Affiliations:** 1 National Clinical Research Center for Mental Disorders (Peking University Sixth Hospital/Institute of Mental Health) and the Key Laboratory of Mental Health, Ministry of Health (Peking University), Beijing, 100191 China; 2 Jiangxi Mental Hospital & Affiliated Mental Hospital, Jiangxi Medical College, Nanchang University, Nanchang, Jiangxi China; 3 The Fourth People’s Hospital of Chengdu, Chengdu, Sichuan China; 4 The Affiliated Brain Hospital, Guangzhou Medical University, Guangzhou, Guangdong China; 5 The Guangxi Zhuang Autonomous Region Brain Hospital, Liuzhou, Guangxi China; 6 Liaoning Provincial Mental Health Center, Tieling, Liaoning China; 7 Shandong Mental Health Center, Shandong University, Jinan, Shandong China; 8 Changchun Sixth Hospital, Changchun, Jilin China; 9 The Second People’s Hospital of Hunan Province (Hunan Provincial Brain Hospital), Changsha, Hunan China; 10 Xi’an Mental Health Center, Xi’an, Shaanxi China; 11 Shanxi Province Mental Health Center, Taiyuan Psychiatric Hospital, Taiyuan, Shanxi China; 12 Beijing Huilongguan Hospital, Affiliated Capital Medical University, Peking University, Huilongguan Clinical Medical School, Beijing, China; 13 Department of Psychiatry, Wuhan Mental Health Center, Wuhan, Hubei China; 14 The Second People’s Hospital of Guizhou Province, Guiyang, Guizhou China; 15 Tianjin Anding Hospital and Mental Health Center of Tianjin Medical University, Tianjin, China; 16 The Sixth Clinical Medical College of Hebei University, Baoding, Hebei China; 17 Hefei Fourth People’s Hospital, Hefei, Anhui China; 18 Chongqing Mental Health Center, Chongqing, China; 19 Inner Mongolia Autonomous Region Mental Health Center, Hohhot, Inner Mongolia China; 20 Suzhou Guangji Hospital, Suzhou, Jiangsu China; 21 Lianyungang Fourth People’s Hospital, Lianyungang, Jiangsu China; 22 The Affiliated Kangning Hospital of Ningbo University, Ningbo, Zhejiang China; 23 Mental Health Center Affiliated to Kunming Medical University, Kunming, Yunnan China; 24 Department of Biostatistics, School of Science, China Pharmaceutical University, Nanjing, Jiangsu China

**Keywords:** Metabolic syndrome, Schizophrenia, Prevalence, Risk profiles, Atypical antipsychotics

## Abstract

**Background:**

Metabolic abnormalities are prevalent among individuals with schizophrenia, contributing to increased medical burden and premature mortality. However, representative data from mainland China remain scarce, particularly within inpatient populations. This study aims to investigate the prevalence and risk profiles of metabolic abnormalities among inpatients with schizophrenia in mainland China.

**Methods:**

We conducted a large-scale, multicenter cross-sectional study, consecutively enrolling adult inpatients with schizophrenia from 218 psychiatric and general hospitals across six major regions of China between January and December 2023. Metabolic abnormalities were defined according to standard clinical criteria for waist circumference, blood pressure, serum lipids, and fasting glucose. Chi-square tests and ordinal logistic regression analyses were used to identify high-risk profiles.

**Results:**

A total of 18,499 valid cases were analyzed, with 84.51% of patients exhibiting at least one metabolic abnormality. The overall metabolic syndrome prevalence was 28.2%, with marked regional variation (17.2%–48.4%). Patients with metabolic abnormalities were more likely to have a later age at onset, co-occurring depressive symptoms, unhealthy lifestyle behaviors, marital history or unemployment, lower educational attainment, and a family history of cardiometabolic disorders. Among commonly used atypical antipsychotics, olanzapine showed the strongest association with metabolic abnormalities (OR = 1.21, 95% CI: 1.04–1.40), whereas ziprasidone showed the weakest (OR = 0.71, 95% CI: 0.54–0.93).

**Conclusions:**

In China, hospitalized patients with schizophrenia exhibit a high prevalence of metabolic abnormalities. Social disadvantage, greater illness burden, and exposure to commonly prescribed atypical antipsychotics characterize high-risk profiles, informing efforts toward early identification and integrated preventive interventions.

**Trial registration:**

Not applicable.

## Introduction

Patients with schizophrenia represent a high-risk population for developing metabolic abnormalities [1]. Among these, metabolic syndrome, characterized primarily by central obesity, dysregulation of glucose and lipid metabolism, and hypertension, may result in reduced treatment adherence and an elevated risk of premature mortality [2]. To enhance patient outcomes, researchers have emphasized prevention-oriented strategies for metabolic management [3]. One prerequisite for developing effective strategies is a comprehensive understanding of the prevalence of metabolic abnormalities, particularly metabolic syndrome, among individuals with schizophrenia [4]. 

Although the prevalence of metabolic syndrome among patients with schizophrenia has been extensively investigated, large-scale studies in mainland China remain limited [5]. It is estimated that more than one-third of individuals with schizophrenia may have metabolic syndrome [6]. However, the prevalence of this condition may vary depending on genetic and environmental factors [7]. Therefore, data derived from other countries may not accurately reflect the metabolic burden of Chinese patients with schizophrenia. Moreover, mainland China has a vast territory and marked demographic diversity, which may contribute to regional variations in prevalence [8]. Particularly, the extent of metabolic risk among inpatients across China remains unclear [9]. 

A deeper understanding of the metabolic risk profile associated with schizophrenia can facilitate the identification of patient subgroups requiring prioritized intervention. In general, the prevalence of metabolic syndrome is higher among individuals receiving antipsychotic treatment compared with those who are not medicated [10]. Even after lifestyle modifications or the initiation of hypoglycemic agents, patients receiving pharmacological treatment may continue to experience persistent metabolic abnormalities [11]. Furthermore, factors such as sex, age, illness duration, type of medication, and length of treatment may influence the development of metabolic abnormalities [12]. However, how these demographic and clinical characteristics contribute to the metabolic risk profile of hospitalized patients with schizophrenia in China remains unclear.

Considering these uncertainties, we collected cross-sectional clinical data on hospitalized patients with schizophrenia through consecutive enrollment from representative psychiatric and general hospitals across various regions of China. The objectives of this study were: (1) to investigate the prevalence of metabolic abnormalities, including metabolic syndrome, among hospitalized patients with schizophrenia in China; and (2) to analyze the demographic and clinical characteristics associated with metabolic abnormalities to identify potential risk profiles. This study aims to provide evidence from a Chinese perspective to support the development of early screening and integrated intervention strategies for metabolic syndrome in patients with schizophrenia.

## Methods

### Participants

This large-scale, multicenter, cross-sectional study was conducted as part of the Prevalence and Risk Profiles of Metabolic Abnormalities (PRIMA) project, targeting hospitalized individuals with schizophrenia in China. From January to December 2023, PRIMA consecutively enrolled participants and collected clinical data from 218 psychiatric and general hospitals across six major geographic regions of China.

Inclusion criteria were as follows: (1) a diagnosis of schizophrenia according to the International Classification of Diseases, 10th Revision (ICD-10); [13] (2) age ≥ 18 years; and (3) provision of written informed consent by the patient and/or their legal representative. Exclusion criteria included: (1) presence of organic brain disorders or severe physical illnesses; (2) a history of psychoactive substance use disorder; (3) a diagnosis of intellectual disability; (4) inability to cooperate due to severe agitation, stupor, or risk of suicide; and (5) pregnancy or lactation.

The study protocol was approved by the Ethics Committee of Peking University Sixth Hospital (Approval No. [2023] Ethics Review No. 39), and written informed consent was obtained from all participants.

### Data collection

A self-developed case report form was used to collect demographic information, clinical characteristics, and laboratory test results, followed by assessment of metabolic risk stratification. Data were managed using the Solutions Electronic Data Capture System (Shanghai, China).

### Basic information collection

Trained research staff collected general demographic data (including date of birth, gender, ethnicity, marital status, employment status, and education level), current medical history (including time of diagnosis, age at onset, disease duration, and medication duration), past medical history (comorbid psychiatric and physical illnesses), smoking and alcohol use history, and family history through face-to-face interviews with participants. The collected information was cross-verified with electronic medical records and family-reported data. In cases of discrepancies, the attending physician determined the more reliable source.

Height, weight, waist circumference, and blood pressure were measured by uniformly trained staff using standardized protocols. Prior to height and weight measurements, participants fasted, removed their shoes, and wore light clothing. Each parameter was measured twice. If the discrepancy exceeded the predefined threshold (height > 0.5 cm, weight > 0.2 kg), a third measurement was obtained, and the average of the two closest values was recorded. Body mass index was calculated as weight divided by the square of height (kg/m²). Waist circumference was measured horizontally 0.5–1 cm above the umbilicus with participants standing in a relaxed posture and gently exhaling. If the difference exceeded 1 cm, a third measurement was taken, and the average of the two closest values was recorded. Systolic and diastolic blood pressure were measured twice on the right upper arm using an electronic sphygmomanometer after at least 5 min of rest, with at least a 1-minute interval between readings. The average value was recorded.

### Clinical characteristics collection

On the day of enrollment, participants were evaluated by uniformly trained psychiatrists using the Clinical Global Impression–Severity of Illness (CGI-SI) and the Calgary Depression Scale for Schizophrenia (CDSS) to assess overall illness severity and depressive symptoms. The CGI-SI was used to evaluate overall severity on a scale from 1 to 7, with higher scores indicating greater severity. The CDSS consists of nine structured items. Each item was scored on a 0–3 scale, with higher scores indicating more severe depressive symptoms. The Chinese versions of the CGI-SI and CDSS are widely used in cross-sectional studies involving Chinese patients with schizophrenia [14, 15]. 

### Collection of laboratory test indicators

Laboratory parameters included fasting triglycerides (TG), high-density lipoprotein cholesterol (HDL-C), and fasting plasma glucose (FPG). On the morning following enrollment (between 6:00 and 7:00 a.m.), fasting venous blood samples were collected at the bedside by trained nurses after ≥ 8 h of fasting. After sample collection, nurses labeled and registered the specimens, which were then transported by designated staff to the clinical laboratories at each site for routine biochemical testing. In cases of markedly abnormal results, retesting or resampling was performed in accordance with standard operating procedures.

### Stratification of metabolic risk populations

Metabolic syndrome (MetS) refers to a cluster of co-occurring metabolic abnormalities. In this study, risk stratification was conducted based on clinical indicators and the diagnostic thresholds recommended in the Chinese Guidelines [16]. Abdominal obesity was defined as a waist circumference ≥ 90 cm for males or ≥ 85 cm for females. Hyperglycemia was identified as a fasting plasma glucose level ≥ 6.1 mmol/L, a 2-hour postprandial glucose level ≥ 7.8 mmol/L, or a confirmed diagnosis of diabetes mellitus under treatment. Hypertension was defined as systolic and/or diastolic blood pressure ≥ 130/85 mmHg or a confirmed diagnosis of hypertension under pharmacological management. Hypertriglyceridemia was determined by TG levels ≥ 1.70 mmol/L. Reduced HDL-C was defined as fasting HDL-C < 1.04 mmol/L.

Individuals who met three or more of the above criteria were classified as having MetS. Those who met one or two criteria were assigned to the borderline metabolic risk group (MetS-B). Participants meeting none of the criteria were classified as the high-vulnerability group (MetS-H), reflecting an underlying susceptibility to metabolic disturbances in schizophrenia rather than current metabolic abnormalities.

### Quality control measures

All participating centers underwent standardized training organized by the project coordination team prior to study initiation. The training covered study protocol interpretation, participant eligibility criteria, scale administration methods, standard procedures for anthropometric and blood pressure measurements, laboratory sample collection and processing, and use of the electronic data capture system.

To ensure consistency in clinical assessments across centers, raters for the CGI-SI and CDSS scales received uniform training to ensure standardized administration. Formal inter-rater reliability testing was not conducted as part of the study protocol. During the study, regular investigator meetings and quality monitoring sessions were held to review rating procedures, address potential inconsistencies, and reinforce standardized assessment practices across sites. Anthropometric and blood pressure measurements were conducted in strict accordance with standard operating procedures, with periodic audits of measurement logs. Deviations were promptly addressed through corrective feedback.

For laboratory testing, all centers used the same brand and model of testing equipment and reagent kits whenever possible and followed unified laboratory protocols. Standardized reference samples were used during the study to assess inter-laboratory and batch-to-batch consistency. Additionally, all data were entered in real time into a centralized electronic data capture system. Quality control personnel regularly monitored missing values and performed cross-site data validation. Suspected anomalies were promptly verified with the corresponding centers and retraining or supplementary data collection was initiated when necessary.

## Statistical analyses

All statistical analyses were conducted using R (version 4.3.3; R Foundation for Statistical Computing, Vienna, Austria) and Stata (version 15.1; StataCorp, College Station, TX, USA). All statistical tests were two-sided, with a significance threshold set at *P* < 0.05.

Demographic characteristics (e.g., age, sex, ethnicity, marital status, employment status, and educational attainment), clinical features (e.g., age at onset, duration of illness, BMI, and duration of treatment), and geographic distribution were compared across three metabolic risk groups. Categorical variables were summarized using frequencies and percentages and compared using chi-square tests. Continuous variables with normal distributions were expressed as mean ± standard deviation (SD) and compared using one-way analysis of variance (ANOVA). Variables with skewed distributions were expressed as median (interquartile range, IQR) and compared using the Kruskal–Wallis test. Normality was assessed using the Shapiro-Wilk test, with non-normal distributions defined as *P* < 0.05.

To further investigate the associations between demographic and clinical characteristics, antipsychotic use, and metabolic abnormalities, a multivariable ordinal logistic regression model was employed, with metabolic risk levels (MetS-H, MetS-B, and MetS) as the ordinal outcome variable. The proportional odds assumption was formally assessed using the Brant test and was not violated, supporting the use of the ordinal logistic regression model. Two separate regression models were constructed: Model 1 included demographic and clinical characteristics as predictors, while Model 2 incorporated antipsychotic medication use. To accurately distinguish and evaluate the associations between individual antipsychotic agents and metabolic abnormalities, individuals were included in Model 2 based on the following criteria: exclusive use of a single antipsychotic agent within the past year, a daily dose equal to or exceeding the World Health Organization’s defined daily dose (DDD), and a treatment duration of at least two weeks [17]. Associations were quantified using odds ratio (OR) and corresponding 95% confidence interval (CI).

## Results

### Demographic and clinical characteristics

Patients with schizophrenia classified as MetS were more likely to exhibit the following demographic characteristics (Table 1): obesity, Han ethnicity, unemployment, being married or divorced, lower educational attainment, and a history of smoking or alcohol consumption. Clinical characteristics included (Table 2): later age of onset, longer illness duration, greater severity, presence of depressive symptoms, history of clozapine/olanzapine use, and family history of hypertension, hyperglycemia, or dyslipidemia.

**Table 1:** Demographic characteristics of patients with schizophrenia across metabolic risk groups

Demographic characteristics	MetS (*n* = 5222)	MetS-B (*n* = 10412)	MetS-H (*n* = 2865)	t/c^2^	*P* value
Gender, n (%)				2.73	0.256
Male	3143 (28.33%)	6274 (56.55%)	1678 (15.12%)		
Female	2077 (28.14%)	4121 (55.84%)	1182 (16.02%)		
Age (year), mean ± SD	44.40 ± 12.29	44.64 ± 13.44	42.54 ± 13.96	62.75	<0.001
BMI (kg/m^2),^ mean ± SD	26.48 ± 3.12	24.33 ± 3.56	21.64 ± 2.80	3386.29	<0.001
Ethnic, n (%)				6.64	0.036
Han	4951 (28.48%)	9758 (56.12%)	2678 (15.04%)		
Ethnic minorities	268 (24.98%)	623 (58.06%)	182 (16.96%)		
Marital status, n (%)				187.98	<0.001
Unmarried	2449 (24.90%)	5627 (57.20%)	1761 (17.90%)		
Married	1992 (32.59%)	3330 (54.48%)	790 (12.93%)		
Divorced	674 (31.99%)	1163 (55.20%)	270 (12.81%)		
Widowed	100 (24.45%)	271 (66.26)	38 (9.29%)		
Employment status, n (%)				79.61	<0.001
Employed	517 (29.01%)	895 (50.22%)	370 (20.76%)		
Unemployed	315 (36.33%)	443 (51.10%)	109 (12.57%)		
Not in labor force	4127 (27.65%)	8550 (57.28%)	2249 (15.07%)		
Retired	257 (29.11%)	494 (55.95%)	132 (14.95%)		
Education level, n (%)				64.57	<0.001
Primary school or below	2238 (30.57%)	4075 (55.65%)	1009 (13.78%)		
Middle and high school	2381 (27.14%)	4946 (56.37%)	1447 (16.49%)		
Junior college	465 (27.35%)	971 (57.12%)	264 (15.53%)		
Bachelor’s degree or above	130 (20.28%)	379 (59.13%)	132 (20.59%)		

Note: MetS = Metabolic Syndrome group (≥ 3 diagnostic criteria met); MetS-B = Borderline group (1–2 criteria met); MetS-H = High-risk group (0 criteria met)

**Table 2:** Clinical characteristics of patients with schizophrenia across metabolic risk groups

Clinical characteristics	MetS (*n* = 5222)	MetS-B (*n* = 10412)	MetS-H (*n* = 2865)	t/c^2^	*P* value
Age at onset of schizophrenia (years), mean ± SD	28.33 ± 9.83	27.69 ± 9.81	27.03 ± 9.55	56.82	<0.001
Illness duration (years), mean ± SD	14.25 ± 10.57	14.83 ± 11.10	13.16 ± 11.56	82.78	<0.001
Antipsychotic use in the past year, n (%)				121.96	<0.001
Olanzapine/Clozapine	3071 (29.91%)	5758 (56.07%)	1440 (14.02%)		
Others	2076 (26.73%)	4332 (55.78%)	1358 (17.49%)		
Treatment duration (years), mean ± SD	11.51 ± 9.69	12.42 ± 10.25	11.23 ± 10.62	67.31	<0.001
CDSS score, n (%)				819.31	<0.001
≥ 6	1814 (46.17%)	1767 (44.97%)	348 (8.86%)		
< 6	3380 (23.43%)	8550 (59.26%)	2499 (17.32%)		
CGI-SI score, mean ± SD	4.00 ± 1.35	3.73 ± 1.43	3.71 ± 1.38	161.93	<0.001
Family history of hypertension, n (%)				9.12	0.010
No	5043 (28.22%)	10,048 (56.22%)	2782 (15.57%)		
Yes	133 (34.64%)	205 (53.39%)	46 (11.98%)		
Family history of hyperlipidemia, n (%)				25.50	<0.001
No	5059 (28.15%)	10,113 (56.27%)	2799 (15.58%)		
Yes	112 (41.03%)	137 (50.18%)	24 (8.79%)		
Family history of hyperglycemia, n (%)				26.08	<0.001
No	5042 (28.15%)	10,086 (56.31%)	2784 (15.54%)		
Yes	120 (41.81%)	133 (46.34%)	34 (11.85%)		
Smoking history, n (%)				106.80	<0.001
Never	3941 (27.07%)	8252 (56.69%)	2364 (16.24%)		
Occasionally	552 (27.95%)	1134 (57.42%)	289 (14.63%)		
Regularly	712 (37.63%)	974 (51.48%)	206 (10.89%)		
Alcohol use history, n (%)				127.84	<0.001
Never	4246 (27.27%)	8820 (56.64%)	2506 (16.09%)		
Occasionally	608 (29.59%)	1168 (56.84%)	279 (13.58%)		
Regularly	337 (44.99%)	351 (46.86%)	61 (8.14%)		

Note: CDSS = Calgary Depression Scale for Schizophrenia; CGI-SI = Clinical Global Impression–Severity of Illness. MetS = Metabolic Syndrome group (≥ 3 diagnostic criteria met); MetS-B = Borderline group (1–2 criteria met); MetS-H = High-risk group (0 criteria met)

### Prevalence of metabolic abnormalities

**Fig. 1:**
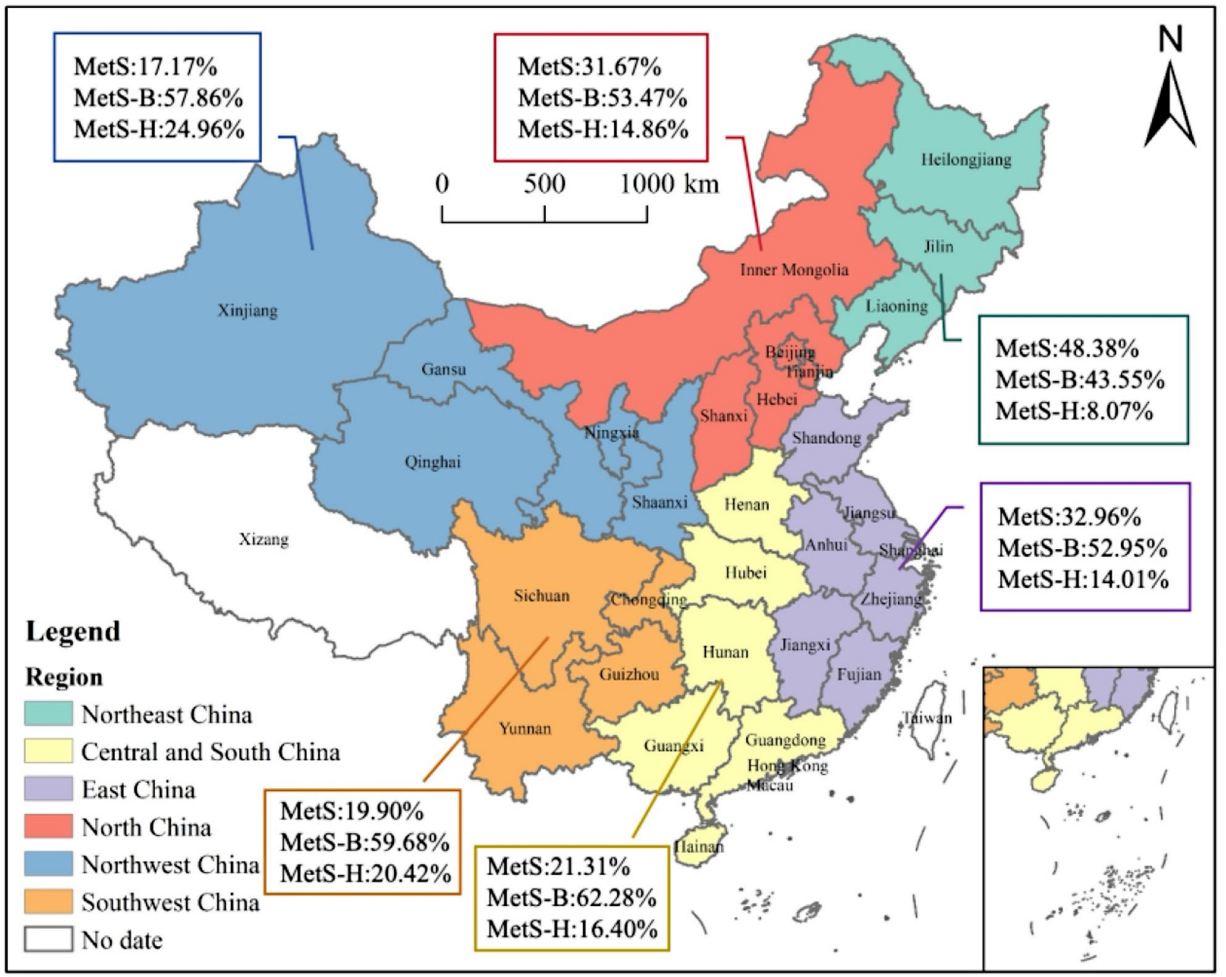
Regional distribution of metabolic risk stratification (MetS, MetS-B, and MetS-H) among hospitalized patients with schizophrenia in mainland China. Note: MetS = Metabolic Syndrome (≥ 3 criteria); MetS-B = Borderline group (1–2 criteria); MetS-H = High-risk group (0 criteria). Base map from https://www.tianditu.gov.cn/

Among hospitalized patients with schizophrenia in China, the overall prevalence of MetS was 28.23%. A total of 84.51% of patients exhibited at least one metabolic abnormality, while only 15.49% presented with no detectable metabolic disturbance. The regional differences in the prevalence of metabolic abnormalities were statistically significant (*P* < 0.001) (Fig. 1).

### Multivariable ordinal logistic regression analysis of factors associated with metabolic abnormalities

**Fig. 2:**
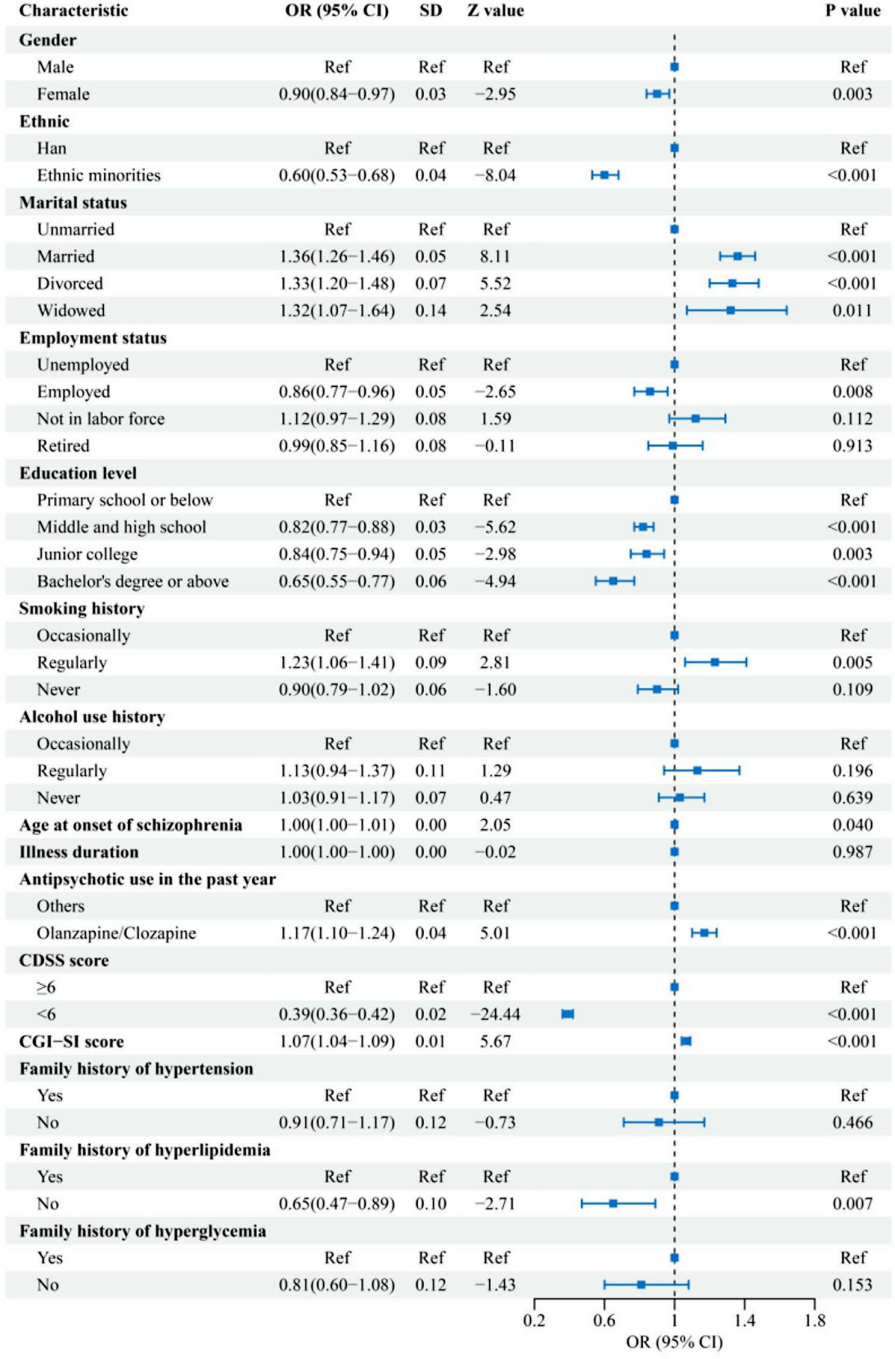
Regression analysis of demographic and clinical factors associated with metabolic abnormalities

Results of Model 1 are presented in Fig. 2. Male sex, Han ethnicity, older age at illness onset, greater illness severity, co-occurring depressive symptoms, history of clozapine or olanzapine use, frequent smoking, adverse marital or employment history, lower educational attainment, and a family history of dyslipidemia were all significantly associated with higher levels of metabolic risk (all *P* < 0.05).

### Association between common antipsychotic use and metabolic abnormalities

**Fig. 3:**
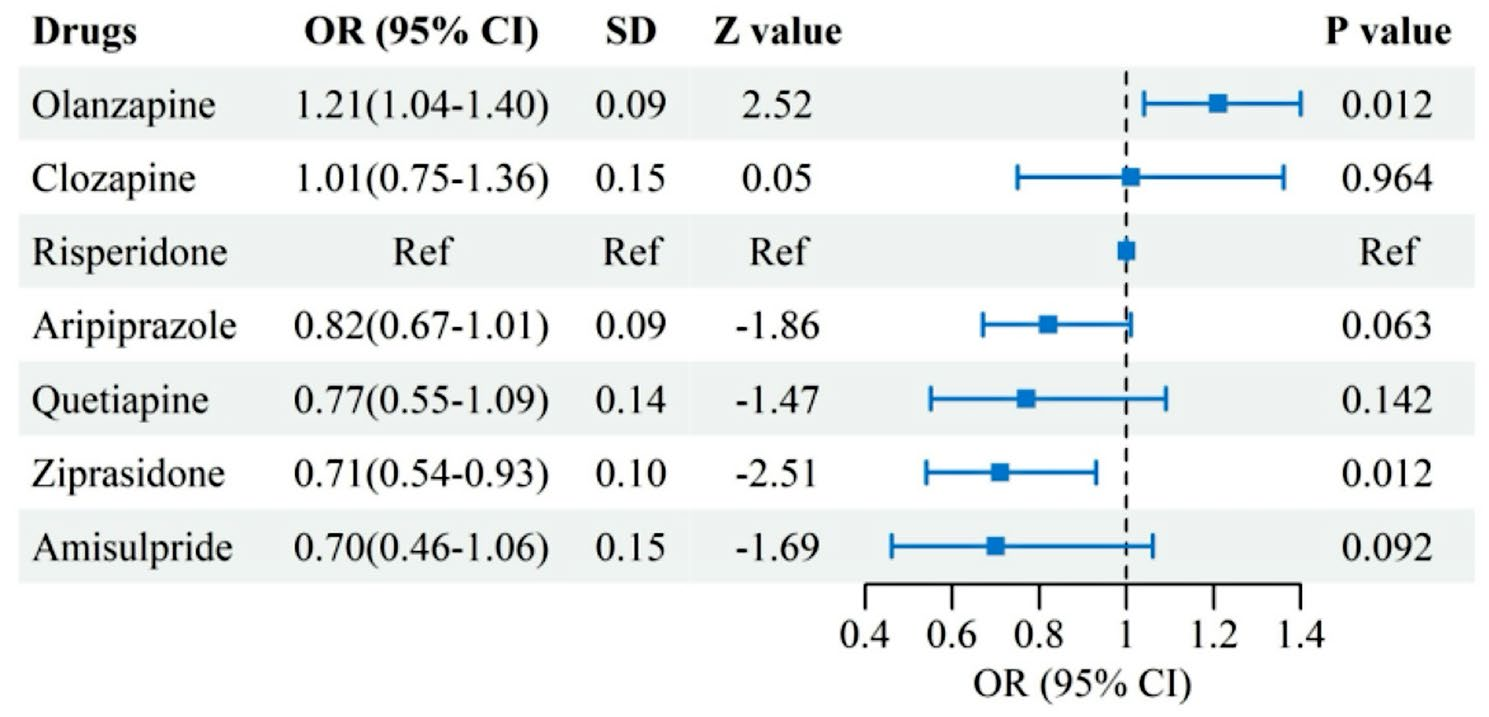
Regression analysis of medication factors associated with metabolic abnormalities

Model 2 included seven commonly prescribed antipsychotics with their defined daily doses (DDDs): risperidone (5 mg), quetiapine (400 mg), olanzapine (10 mg), amisulpride (400 mg), clozapine (300 mg), aripiprazole (15 mg), and ziprasidone (80 mg), with risperidone specified as the reference antipsychotic. Four additional antipsychotics (perospirone, blonanserin, paliperidone, and lurasidone) were excluded due to insufficient sample sizes for robust statistical estimation. A total of 5,795 individuals were included in Model 2. The model was adjusted for main confounders, including medication dose, treatment duration, sex, ethnicity, age at illness onset, marital status, employment status, and educational attainment. Results are presented in Fig. 3. Using risperidone as the reference antipsychotic, olanzapine showed the strongest association with metabolic abnormalities (OR = 1.21, 95% CI: 1.04–1.40), whereas ziprasidone showed the weakest association (OR = 0.71, 95% CI: 0.54–0.93), indicating a lower relative association compared with risperidone rather than a negative or protective effect.

## Discussion

### High prevalence of metabolic syndrome among Chinese inpatients with schizophrenia

The overall prevalence of MetS among patients with schizophrenia in China was 28.23%. Compared with previously reported prevalence rates in other Asian countries, the rate in China was comparable to that in Japan (27.5%) and lower than in South Korea (36.5%) [18, 19]. Besides, 84.51% of patients had at least one abnormal metabolic indicator, suggesting that metabolic disturbances may be highly prevalent among patients with schizophrenia, irrespective of medication status [20]. Atypical antipsychotics have been shown to promote lipid synthesis even in the early stages of treatment, prior to observable weight gain [21]. The progression of weight gain and dyslipidemia may contribute to insulin resistance, eventually leading to diabetes mellitus and the fulfillment of multiple diagnostic criteria for MetS [22]. Therefore, understanding the prevalence of metabolic abnormalities is crucial for assessing the patient burden.

The marked geographic variability in the prevalence of metabolic abnormalities may partly reflect regional differences in socioeconomic context and healthcare system characteristics, even within a hospitalized population [23]. Socioeconomic disparities across regions may influence long-term lifestyle patterns, dietary structure, and cumulative metabolic risk prior to hospitalization [23]. In addition, regional variation in psychiatric service organization, hospitalization thresholds, and patient case-mix, including illness chronicity and treatment history, may contribute to differences in metabolic profiles at admission [1, 23]. Furthermore, differences in antipsychotic prescribing patterns and treatment strategies across regions may further shape the observed geographic heterogeneity [24]. 

### Social disadvantage and a greater illness burden characterize high-risk profiles

We found that unemployment, marital disruption, and low educational attainment were significantly associated with metabolic abnormalities. Previous reviews have indicated that, irrespective of national development level, MetS is more prevalent among individuals with low education, low income, unskilled occupations, and broader socioeconomic disadvantage [25]. Longitudinal cohort studies have further suggested that social disadvantage during adolescence may increase the risk of MetS in adulthood through sustained exposure to material and social adversity [26, 27]. Moreover, frequent smoking or alcohol consumption, which are unhealthy lifestyle behaviors more commonly observed in socially disadvantaged populations, may contribute to an elevated risk of metabolic abnormalities [28]. Mechanistically, psychosocial stress resulting from social disadvantage may influence health behaviors and increase cardiovascular risk through activation of neuroendocrine pathways, thereby contributing to the development of MetS [28]. 

This study also identified several markers of higher disease burden that may contribute to the development of metabolic abnormalities. Obesity was highly prevalent among patients with MetS and is not only a diagnostic component of the syndrome but also a driver of insulin resistance, dyslipidemia, and hypertension [29]. Furthermore, clinical features associated with poor schizophrenia prognosis, including late age of onset, prolonged illness duration, greater severity, and comorbid depressive symptoms, were more common among patients with MetS [30, 31]. These features impair both social functioning and overall health, increasing the risk of metabolic complications [31]. Importantly, MetS itself contributes to poor clinical outcomes by reducing treatment adherence and increasing the risk of relapse, thereby creating a vicious cycle of worsening metabolic dysfunction [32]. Finally, patients with a family history of hypertension, hyperglycemia, or dyslipidemia had a significantly higher prevalence of MetS, supporting a role for genetic predisposition in the pathogenesis of MetS [33]. 

### Attention to drug selection and prevention strategies during antipsychotic treatment

Except for ziprasidone, commonly prescribed antipsychotics were all associated with varying degrees of risk for metabolic abnormalities. Although the effect sizes observed for individual antipsychotics were modest, likely in part due to the use of risperidone as an active comparator, these associations may still carry clinical relevance when considered in the context of long-term treatment and population-level metabolic risk. A longitudinal study on antipsychotic-induced metabolic changes in Chinese patients reported that olanzapine, quetiapine, and risperidone significantly increased the risk of MetS compared to ziprasidone [34]. Aripiprazole was also found to be associated with metabolic abnormalities in this study, aligning with previous research conducted on first-episode schizophrenia patients in China [35]. Although some studies have suggested that amisulpride may have slight advantages over risperidone in terms of weight gain or prolactin elevation [36], our findings indicate that the difference may not be clinically meaningful. Interestingly, clozapine did not demonstrate a higher metabolic risk compared to risperidone, which is consistent with previous findings in Asian populations [37]. One possible explanation is that ethnic differences in single nucleotide polymorphisms may influence susceptibility to clozapine-induced MetS [38, 39]. 

From a clinical perspective, these findings should not be interpreted as supporting rigid antipsychotic selection based solely on metabolic risk estimates, particularly given the modest effect sizes observed in comparisons relative to risperidone, a drug already associated with metabolic abnormalities. Instead, the results underscore the importance of individualized risk stratification and proactive metabolic monitoring during antipsychotic treatment [40]. When high-risk agents such as olanzapine or clozapine are required, they may be used in combination with metabolic protective agents such as metformin, alongside early lifestyle interventions and regular metabolic monitoring [41]. Alternatively, metabolically favorable agents such as ziprasidone may be considered as first-line options or in combination therapy to reduce the metabolic burden in patients with schizophrenia [42, 43]. 

## Limitations

Although this study used a large cross-sectional sample from six major regions of China, providing substantial geographic representativeness, several limitations should be acknowledged. First, the cross-sectional design limits causal interpretation, and observed associations between antipsychotic use history and MetS should not be interpreted as causal. Metabolic abnormalities may precede antipsychotic exposure in some patients, raising the possibility of reverse causality or indication bias. Second, drug exposure data were derived from retrospective clinical records. Although defined daily dose thresholds and minimum duration criteria were applied to improve classification accuracy, actual cumulative dose and treatment duration were not quantified, which may have led to underestimation of dose-dependent metabolic effects.

Third, antipsychotic polypharmacy was not explicitly quantified in the current analyses, which may have influenced the observed metabolic risk profiles, particularly in this hospitalized population where polypharmacy is relatively common. Combination antipsychotic treatment is often initiated to enhance symptom control or manage treatment resistance and has been associated with an increased metabolic burden in some patients. Conversely, in certain clinical contexts, antipsychotic combinations may be selected or adjusted with the aim of mitigating metabolic adverse effects. As a result, the net impact of antipsychotic polypharmacy on metabolic risk may be heterogeneous and bidirectional, and could not be fully disentangled in this study.

Fourth, although multiple demographic and clinical confounders were adjusted for in the regression models, residual confounding from unmeasured factors cannot be fully excluded. Key determinants of metabolic health, such as dietary patterns, physical activity levels, and sedentary behavior, were not directly measured in the present study and may independently influence metabolic outcomes. In addition, socioeconomic factors and health-related behaviors may shape long-term metabolic risk trajectories prior to hospitalization. The inability to account for these variables may have contributed to the observed associations and should be considered when interpreting the findings. Future studies incorporating detailed lifestyle and behavioral measures may help further clarify the extent of residual confounding and refine risk estimation.

Moreover, information on non-psychotropic medications was not collected in this study. In particular, the use of antidiabetic agents or weight loss medications, which may influence metabolic parameters, was not accounted for in the analyses. The absence of these data may have introduced residual confounding and should be considered when interpreting the associations between antipsychotic use and metabolic abnormalities.

Fifth, although schizophrenia is characterized by prominent psychotic symptoms, detailed symptom-level assessments such as the Positive and Negative Syndrome Scale (PANSS) were not collected in the present study. This decision was primarily driven by feasibility considerations in a large, hospitalized, multi-center sample, as PANSS administration is time-consuming and requires extensive rater training to ensure inter-rater reliability. Instead, overall illness severity was assessed using the CGI-SI, which has been widely applied as a pragmatic indicator of global clinical status. Nevertheless, the absence of detailed psychotic symptom measures may represent a source of residual confounding and should be considered when interpreting the findings.

In addition, the clinical rating scales used in this study, including the CGI-SI and CDSS, are inherently subjective and may be subject to inter-rater variability. Although standardized training was provided to all raters, formal inter-rater reliability metrics (e.g., intraclass correlation coefficients) were not calculated, which may have introduced measurement variability. This limitation should be considered when interpreting associations involving these clinical ratings.

Sixth, some laboratory parameters were measured independently at local sites. Although unified quality control procedures were applied across centers, inter-site variability in instruments and reagents may have introduced measurement bias. Finally, because the sample primarily comprised hospitalized patients with relatively severe illness, the overall prevalence of metabolic abnormalities may have been overestimated, limiting the generalizability of the findings to the broader schizophrenia population. Besides, diagnostic criteria and parameter cut-offs for metabolic syndrome vary across different guidelines, including the Chinese guideline, the World Health Organization, the American Heart Association, and the International Diabetes Federation [44]. The use of different definitions may influence prevalence estimates and limit direct comparability across studies. Therefore, the findings should be interpreted within the context of the diagnostic criteria applied in the present study.

## Conclusion

Based on multicenter cross-sectional data from six major regions in China, this study systematically evaluated the prevalence of metabolic abnormalities and its risk profiles among hospitalized patients with schizophrenia. The findings revealed a relatively high prevalence of metabolic syndrome among patients with schizophrenia, with marked regional disparities. Social disadvantage and higher disease burden may contribute to an elevated risk of metabolic abnormalities in this population. In addition, the use of commonly prescribed antipsychotics was associated with metabolic abnormalities. Future efforts should prioritize the early identification of metabolic abnormalities in patients with schizophrenia and consider high-risk profiles to optimize treatment strategies.

## Data Availability

The datasets used and/or analysed during the current study are available from the corresponding author on reasonable request.

## Abbreviations

MetS: Metabolic Syndrome
MetS-B: Metabolic Syndrome, borderline group
MetS-H: Metabolic Syndrome, high-risk group
CGI-SI: Clinical Global Impression–Severity of Illness
CDSS: Calgary Depression Scale for Schizophrenia
TG: Triglycerides
HDL-C: High-Density Lipoprotein Cholesterol
FPG: Fasting Plasma Glucose
BMI: Body Mass Index
DDD: Defined Daily Dose
